# Temporal and spatial characteristics of sediment sources on the southern Yangtze Shoal over the Holocene

**DOI:** 10.1038/s41598-018-33757-5

**Published:** 2018-10-22

**Authors:** Chao Cao, Feng Cai, Yongling Zheng, Chengqiang Wu, Huiquan Lu, Jingjing Bao, Quan Sun

**Affiliations:** Third Institute of Oceanography, State Ocean Administration, Xiamen, 361005 China

## Abstract

The sediment sources of the Yangtze Shoal were traced by analysing surface and core sediment particle size, detrital and clay minerals, carbon and nitrogen isotopes, and radioisotope dating. In the estuary, the sediments are dominated by silty clay, high stable mineral, and extremely high illite/chlorite. Stable organic carbon isotopes (*δ*^13^C-TOC) indicated a marine-dominated mixture. On the shoal, the sediments are mainly composed of fine sand, high unstable mineral. The *δ*^13^C-TOC indicated predominantly marine sedimentation. The average TOC of core sediments was ~0.26%, and the average TN was ~0.05%. The TOC/TN was 5.4–7.8, the *δ*^13^C-TOC was −19.8 to −22.1‰, and the age of the sediments spanned the last ~10.8 ka (Holocene). The sediments and provenance of the Yangtze Shoal have been controlled by the East Asian monsoon, sea level change, riverine sediment flux and ocean circulation. The intervals 8.3–6.3 ka and 3.8–1.5 ka, are characterized by Yangtze River sources, whereas 6.3–3.8 ka and 1.5–0.8 ka, are characterized by a source mixture with Yellow River input. Tracing the multiple sources effectively confirms the hypothesis that the southern Yangtze Shoal was a delta formed by combined sedimentation from the Yangtze River and Yellow River during times of low sea level.

## Introduction

**Figure 1:**
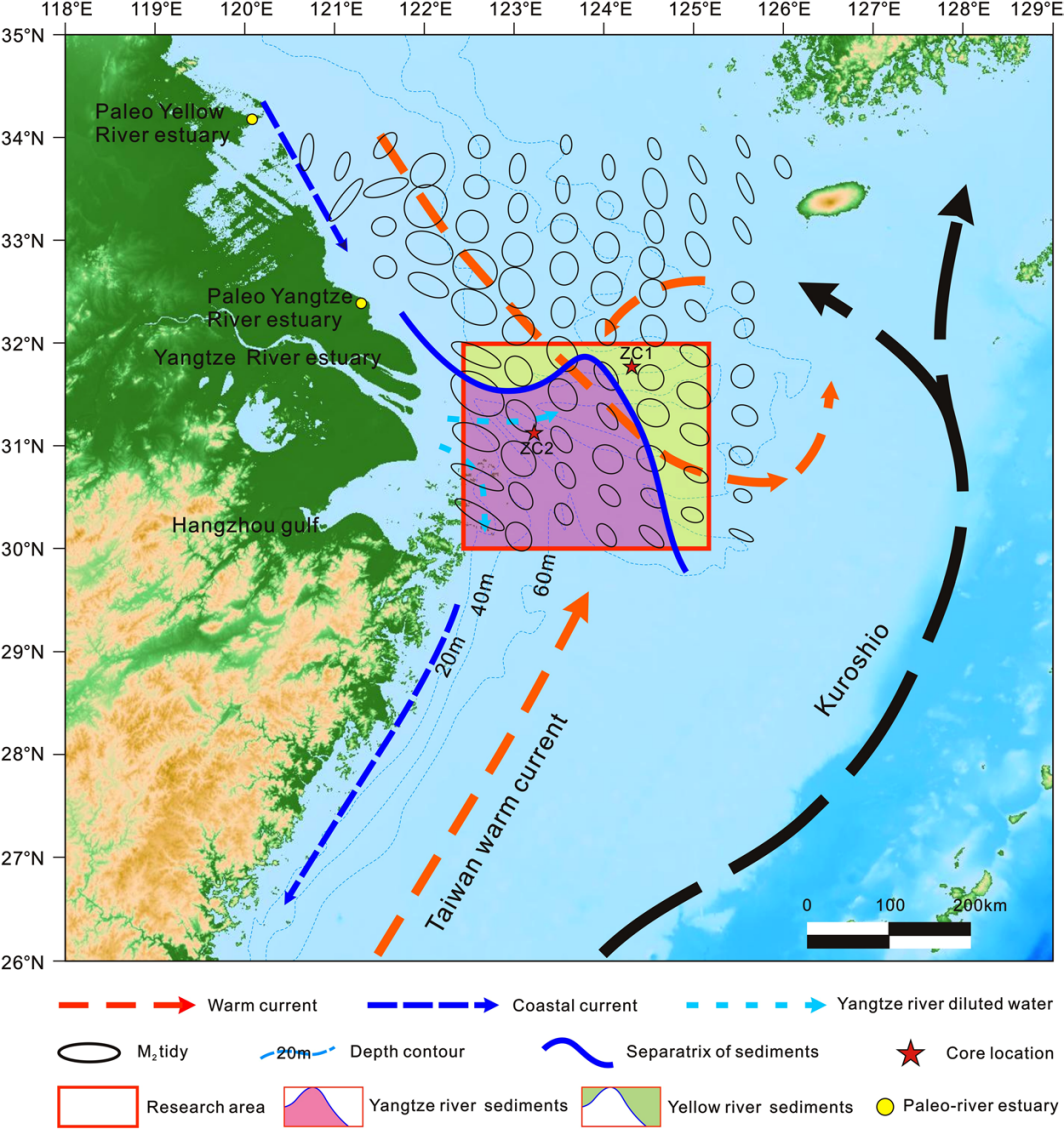
Location maps of research area. Two red stars respresent the sedimentary core site, where distributed different from areas by Yellow River source and Yangtze River source. Flow field data modified after Fan (2002). The map was created with ArcGIS 10.3.

The Yangtze Shoal, the largest in the Yangtze Estuary with an area of 3 × 10^4^ km^2^, is located on the estuary’s eastern side^1,2^. It is a huge sandy sedimentary body that opens to the northwestern Pacific Ocean^3^. The Yangtze Estuary receives a large amount of terrigenous material from the Yangtze River and Yellow River. Tidal currents from the northwestern Pacific, including warm currents from the Yellow Sea and around Taiwan, and fresh water input from the Yangtze River produce sand mats with two grooves and three ridges that comprise the shoal (Fig. 1)^4–7^. However, many questions about its origin, sources and evolution remain, including where does the material for such a huge deposit come from and what are the distribution patterns of this material? There is currently no unified understanding. Some studies suggested that the Yangtze Shoal originates from residual terrestrial sand of the combined paleo-Yangtze and Yellow River delta, and that the Holocene sediments from the shallowest area were removed^8–10^. Other studies suggested that it is sandstone desertification of paleo-structured terraces and a high speed accumulation body formed under storm action^11,12^. Finally, there is also the hypothesis that the Yangtze Shoal originated from interactions between the modern Yangtze River and the ocean, which formed a coarse-grained residue under modern hydrodynamic washing^13,14^.

Sediment grain size composition and parameters can describe characteristics of the sedimentary environment and sediment transport processes^15–20^. Marine sedimentary clay minerals track changes in ocean currents, and their vertical distribution is interpreted broadly as reflecting terrestrial climate change in the provenance area^21,22^. Detrital minerals can trace the source–sink process, which significantly helps to reveal the origin of marginal sea sediments and Quaternary paleo environmental changes^23–25^.

**Figure 2:**
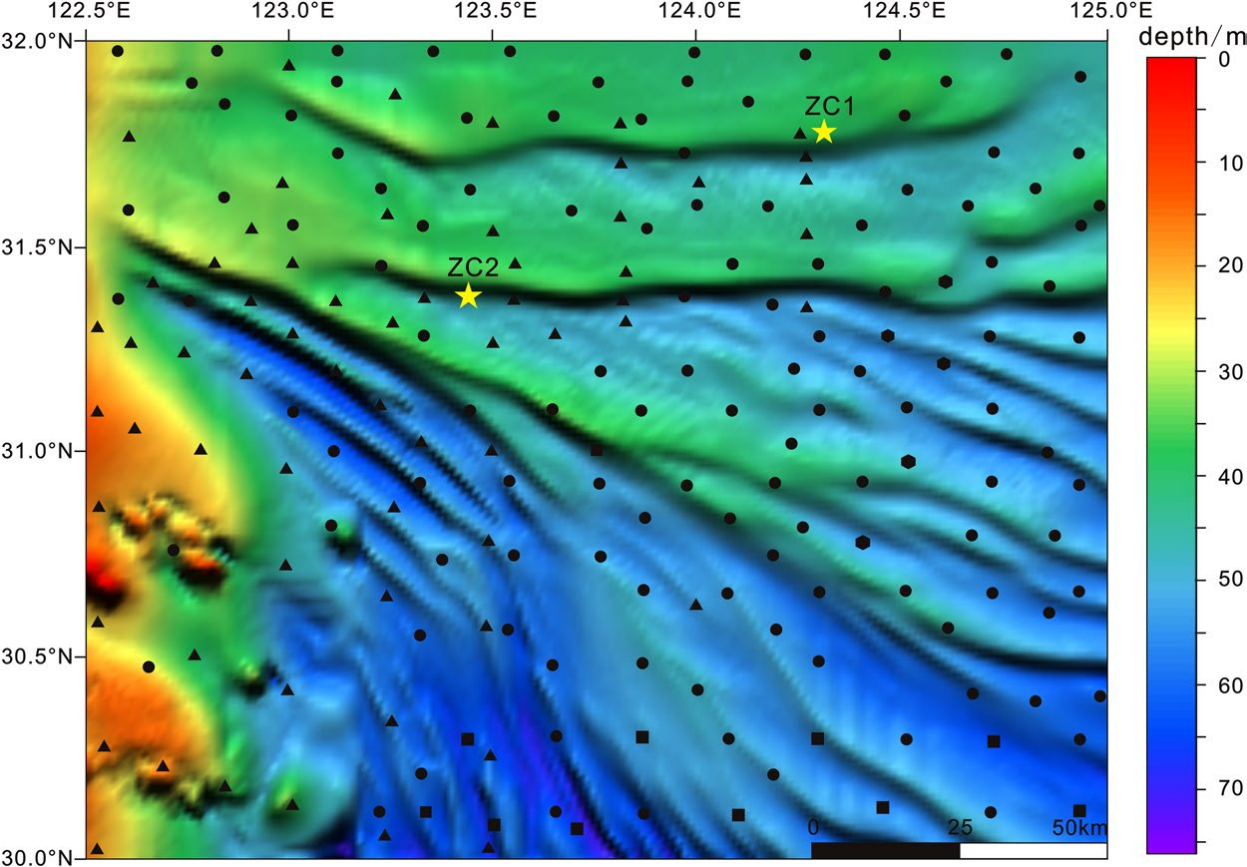
Distribution of sediments on Yangtze Shoal (Cai *et al*.^26^). The research area is about 3 × 10^4^ km^2^. Core ZC1 distributed on the sand ridge. Core ZC2 distributed on the sand groove. All surface samples were obtained in 2009 (), 2013 (), 2014 () and 2015 (), and core samples were obtained in 2014 ().

In this study (Fig. 2), sediment distribution patterns, their sources and types were determined by analysing particle size, detrital and clay minerals, and carbon and nitrogen isotope composition, as well as constructing a chronology with radio isotopic dating. This information was used to describe sedimentary environments during the Holocene and provide basic evidence for the fine landforms and genetic mechanisms of the southern Yangtze Shoal.

## Results

### Granularity distribution characteristics of surface sediments

**Figure 3:**
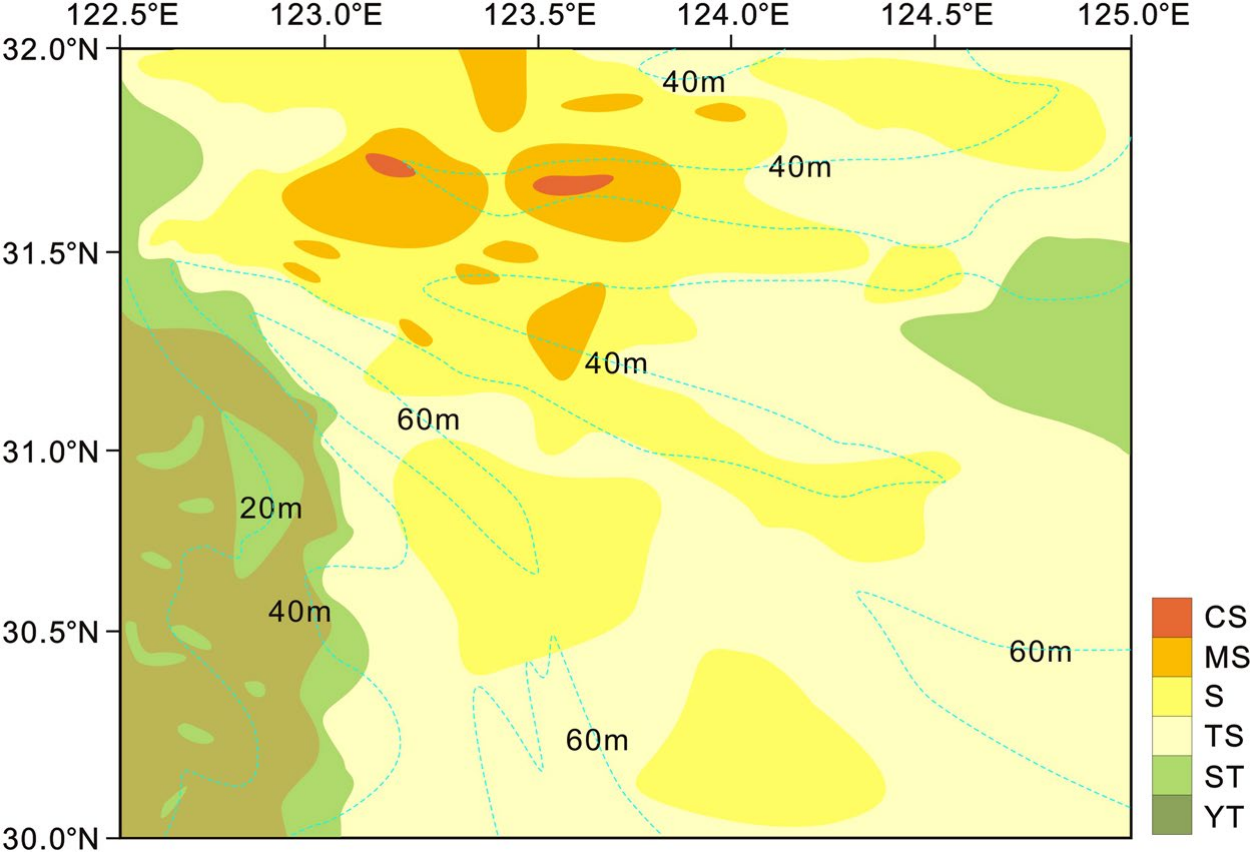
Surface sediment type distribution map of the southern Yangtze Shoal. Sediment type is accroding to sheppard classification (Sheppard, 1954). The light blue dashed line is the isobath line.

The surface sediment grain sizes were within 2–8 Φ. The sediments in the northern area were coarser, with diameters of 3–5 Φ. The sediments on the southern Yangtze Shoal were finer (6–8 Φ). In general, the trend was finer grain sizes close to the Yangtze River estuary that gradually coarsened further offshore, and tapered beyond 124.5°E. According to the Shepard classification criteria, the surface sediments on the southern part of the Yangtze Shoal are characterized by coarse sand, middle sand, fine sand, silty sand, sandy silt and clay silt (Fig. 3). Fine sand is the main type of sandy sediment on the East China Sea shelf. Over 65% of the surface sediments in the study area are sandy sediments. The sedimentary strata of the shoal are based on fine sand and sandy silt, with rhythmic alternations. The sandy sediments at the shelf edge often contained large volumes of intact shells or biological debris. The sediments types in the study area were distributed in regional, zonal and patchy patterns, which were constrained by the terrain^26,27^.

### Detrital minerals distribution characteristics of surface sediments

**Figure 4:**
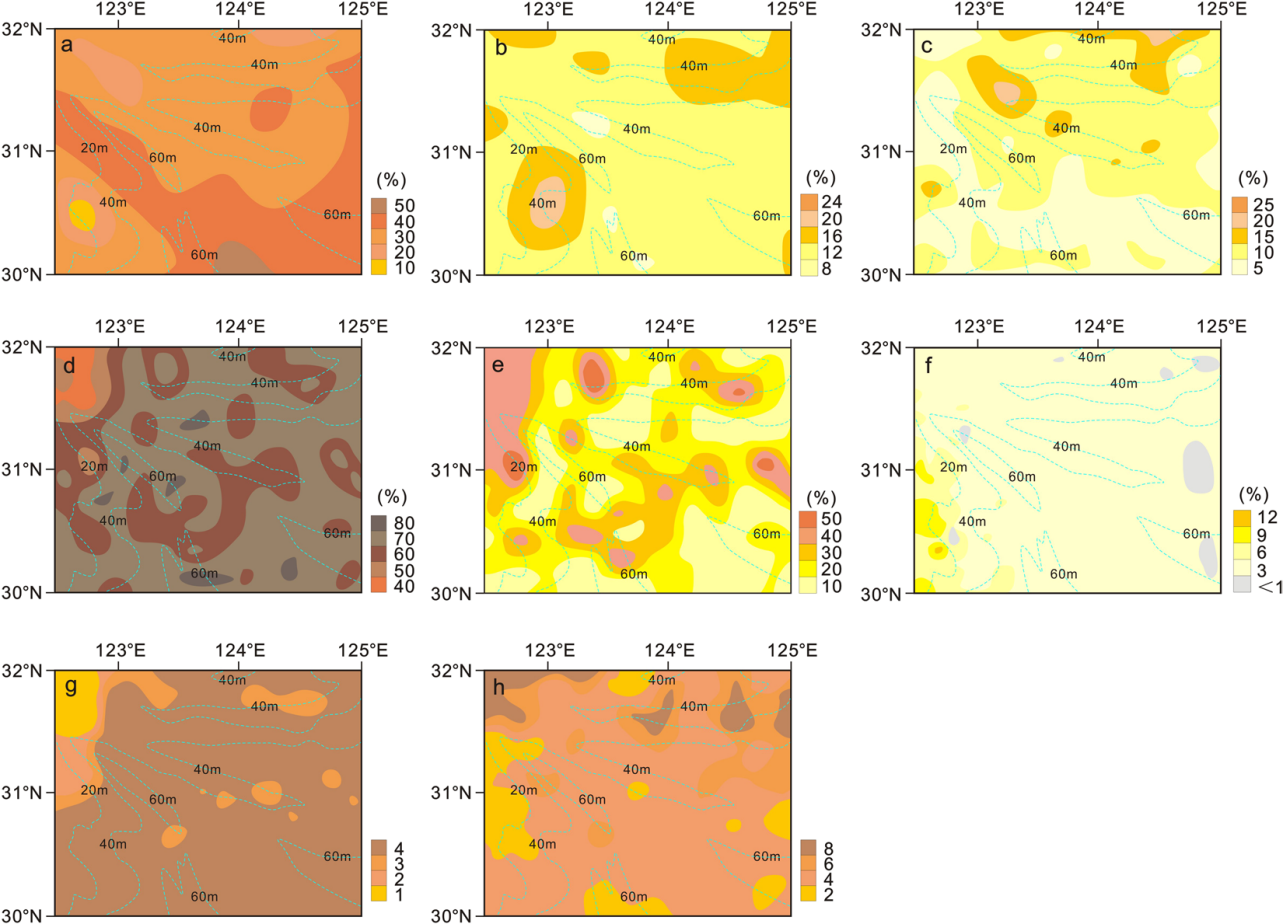
Distribution characteristics of detritus mineral on the southern Yangtze Shoal. Hornblends (**a**), epidote (**b**), metal mineral (**c**), quartz (**d**), feldspar (**e**) and mica (**f**) are selected as typical detritus mineral on research area. Percentage of mass indicates detritus mineral content. Ratio of unstable minerals to stable minerals (**g**) and feldspar to quartz (**h**) are selected as typical material source. Unstable minerals includes hornblendite, augite and olivine in this research. Stable minerals includes zirconite, apatite, titanomagnetite and biotite in this research. The light blue dashed line is the isobath line.

In the surface sediments, the most common amphiboles were green, light green and dark brown in colour, mostly short columns, granular and abrasive; they had an average content of 35.1% and ranged between 22.1–48.9% (Fig. 4a). The highest content was mainly in the Yangtze River estuary, in the eastern Hangzhou Bay and the outer continental shelf; its content in the middle part of the outer continental shelf tended to increase gradually to the south. The distribution of ordinary amphiboles and other heavy minerals showed sediment homogeneity between the East China Sea shelf and the Yangtze River estuary^28^.

The dominant heavy mineral, epidote (8.3–23.3%, average 17.5%), is yellow-green, sub-angular, translucent, granular-based, weathered, and mostly altered from pyroxene and amphibole (Fig. 4b). The highest content area was in the northern beach ridge region. The general distribution of epidote was lower in the nearshore area and increased seaward.

Metallic minerals included hematite, limonite, ilmenite and magnetite. Hematite is a black or dark iron oxide and mostly granular. Limonite exists in cryptocrystalline minerals. Ilmenite and magnetite are mostly granular or irregularly granular, bright black with a strong metallic lustre; the majority of angular edges are abrasive in the sea. The average metallic mineral content was 12.8% and the maximum content was 25.1% (Fig. 4c). The area with high content was mainly on the northeastern part of the main shoal; in general, contents were higher in the north, lower in the south, with a zonal distribution from west to east. The source of the minerals and the weathering intensity of the sediments in the sea play a controlling role in the distribution of metallic minerals.

The main features of quartz morphology were granular, sub-angular and sub-round, with partial abrasion. The average content was 56.7%, and ranged from 46.7–84.3% (Fig. 4d), its average content was higher than feldspar. Quartz overall showed a patchy distribution with low content nearshore and higher content on the middle of the shoal, indicating a strong hydrodynamic environment.

Feldspars included potassium feldspar and plagioclase. Potassium feldspar was mostly red, brown, light brown, granular and hard. Plagioclase appeared granular and yellow, grey and green under the stereoscope. The surface was cloudy, light dim colour with erosion. Feldspars were widely distributed on the Yangtze Shoal, with an average content of 29.1% and a range of 19.7–41.7% (Fig. 4e). The area with high content was near the mouth of the estuary and the tidal current ridge. Its distribution indicated a Yangtze River source and the characteristics of material transportation and proliferation.

Mica species (light minerals) were widely distributed, with an average content of 5.4%, ranging from 0.2–12% (Fig. 4f). The area with high content is located in the southern coastal waters near the shoal, with a gradual reduction seaward.

The radio of unstable mineral to stable mineral was from 1 to 4 (Fig. 4g). The stable mineral mainly distributed in Yangtze Estuary, and the radio in most other area was high. The radio of feldspar to quartz was from 2 to 8 (Fig. 4h). The high radio appeared on the groove.

### Clay minerals distribution characteristics of surface sediments

The study area is situated in the mid-latitudes and within a subtropical climatic zone; thus, chemical weathering is not extensive. The clay minerals remain in the stage of de-potash. Within the clay mineral assemblage, illite content was highest, followed by chlorite and kaolinite, and smectite had the lowest abundance.

**Figure 5:**
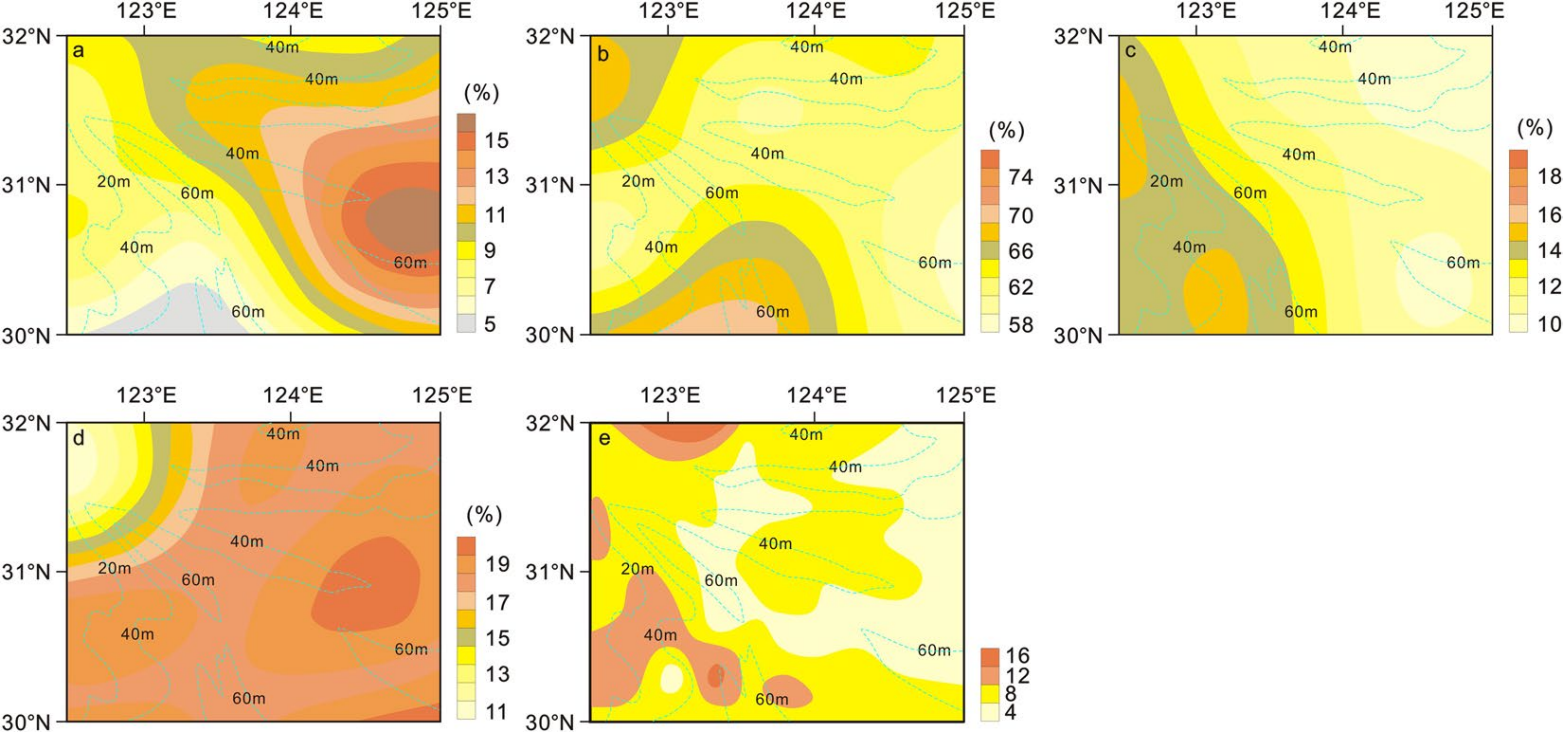
Distribution characteristics of clay mineral on the southern Yangtze Shoal. Smectite (**a**), illite (**b**), kaolinite (**c**) and chloritev(**d**) are selected as typical clay mineral on research area. Percentage of mass indicates clay mineral content. Ratio of smectite to illite (**e**) is selected as typical material source. The light blue dashed line is the isobath line.

The average content of smectite is 8.6% and its content ranged from 5.1–15.9% (Fig. 5). The content of smectite gradually increased from west to east, with the highest values in the southeastern area of extensive ocean. The average content of illite was 67.8%, and the content ranged between 58.2% and 76.9%. The illite content showed a regional distribution, with low content on the northern and outer shoal was low and high values nearshore and in the southern waters. The average content of kaolinite was 13.4%, and ranged from 10.0–19.9%. The kaolinite gradually increased from southwest to northeast and exhibited a zonal distribution. The average content of chlorite was 17.6%, ranged from 10.6–20.4%; its distribution was similar to illite.

The clay minerals in sediments near the Yangtze River estuary were mainly derived from terrestrial material carried by the Yangtze River and deposited under the combined influence of the Yangtze River outflow and coastal currents. The Yellow River material carried along the northern coast also contributed to some extent^29,30^.

**Figure 6:**
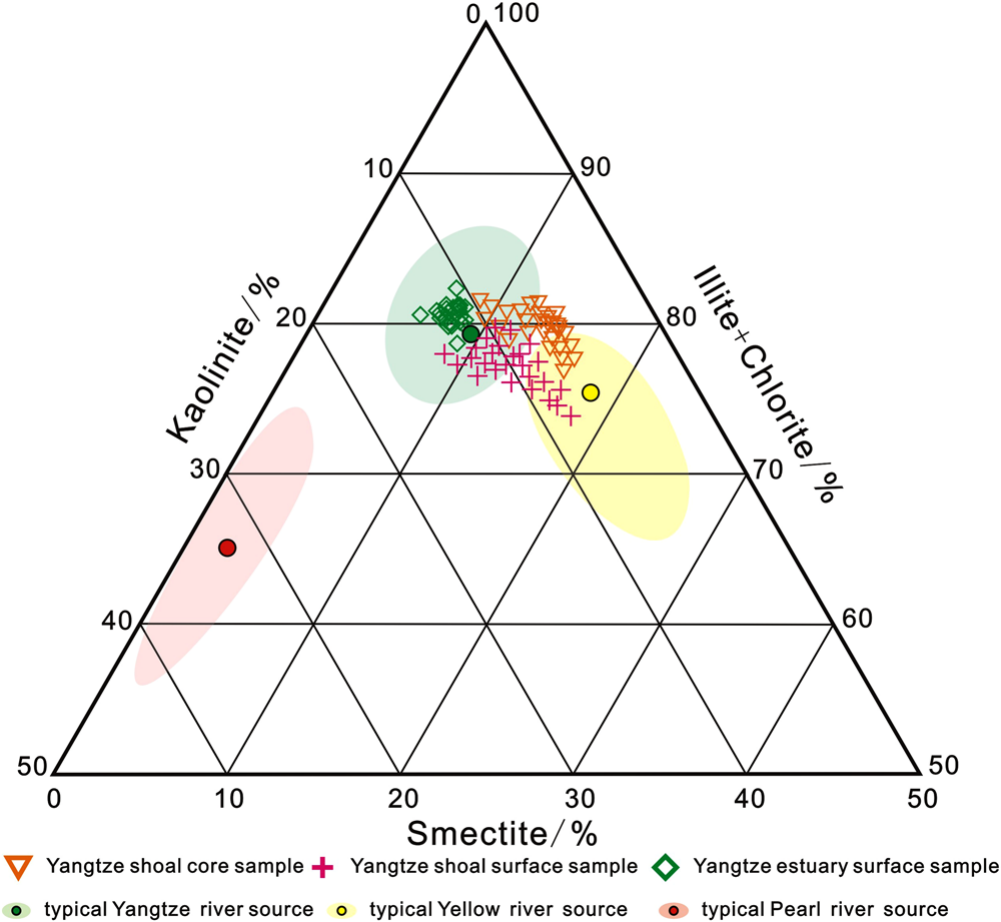
Temary diagram of major clay mineral of sampling sediments. Ternary diagram of the major clay mineral groups illite + chlorite, kaolinite, and smectite. Percentage of mass indicates clay mineral content. Seafloor surface samples and core samples. Shaded areas showing two provinces (Yangtze River source and Yellow River source) of clay mineral assemblages in surface and core samples. Typical Yangtze River source and Yellow River source refer to Liu^27^, and typical Pearl River source refers to Liu^28^.

According to the distribution of clay minerals (Fig. 6), the sediments in the Yangtze River have an illite content of ~70–75%, a smectite content of ~5–8%, and a ratio of illite/smectite >8 (Fig. 5e). The illite content of Yellow River sediments was <60% and smectite content was ~15–20%, the illite/smectite ratio was <6^31–33^. About 40% of the shallow sediments on the southern Yangtze Shoal are located in the source region of the Yellow River, while the other 60% are in the Yangtze River source region. This indicates that the Yangtze River input controls the composition of sand and tidal sediments throughout the study area with the Yellow River also contributing material to this area to a lesser extent.

### Sediment core characteristics

**Figure 7:**
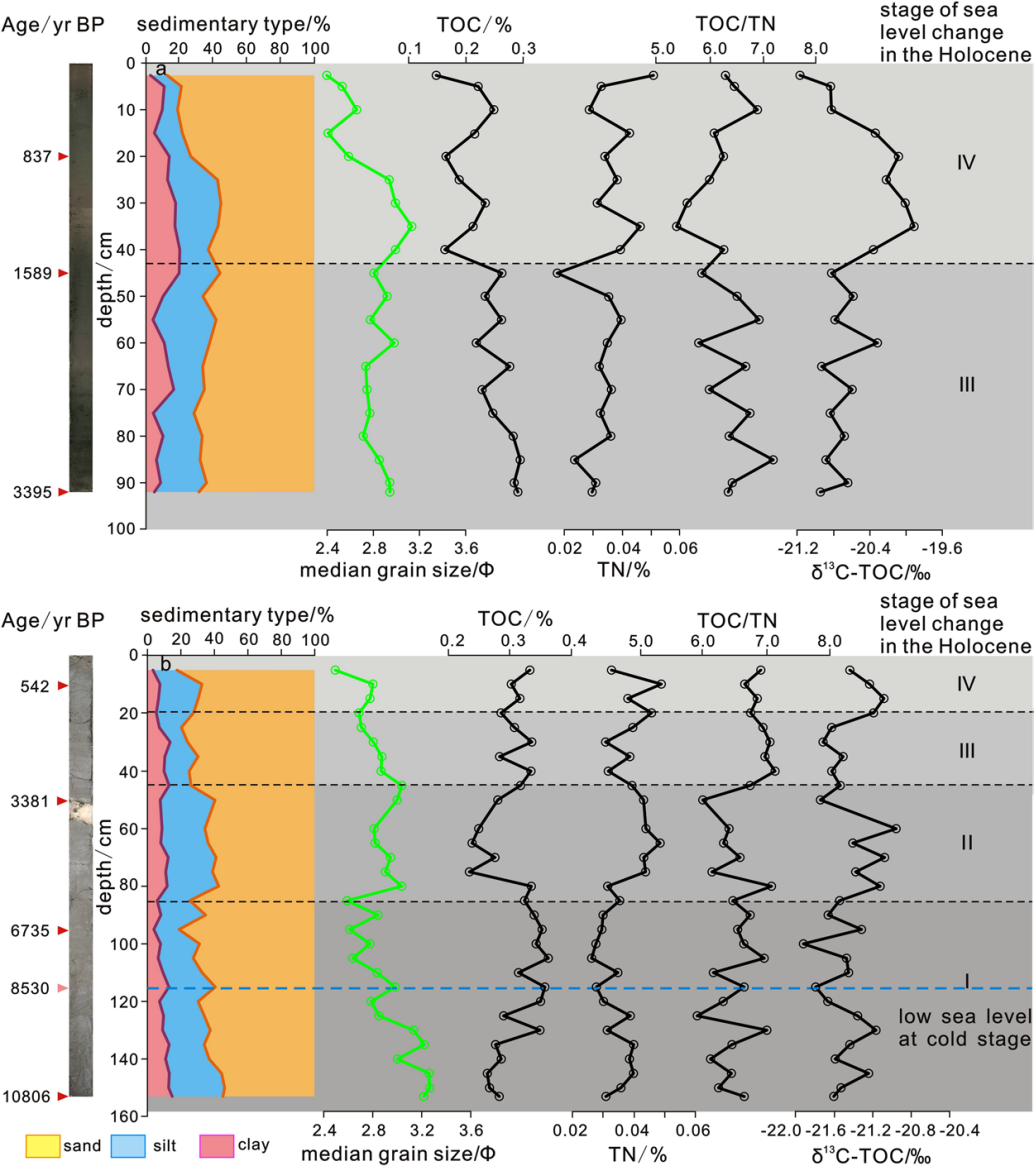
Integrated profile of Core ZC1 (**a**) and ZC2 (**b**). Core ZC1 is on the troughs of the northern part on the southern Yangtze Shoal. The age at the base of core ZC1 is 3395 yr B.P., indicating late Holocene strata. Core ZC2 is on the grooves of the southern part on the southern Yangtze Shoal. The age at the base of core ZC2 is 10806 yr B.P., The sedimentary type, average particle size, TOC, TN, TOC/TN and *δ*^13^C-TOC are showed in this profile diagram. The red triangle shows the measured age, the pink triangle shows the conjectural age.

Core ZC1 was recovered from the area south of the Yangtze Shoal at the top of the sand ridge on the northern part of the shoal. The core depth extended 96 cm (90 cm under testing). The sediments were grey with black organic matter in some layers. The sediment median particle size was 2.5–4.0 Φ (Fig. 7a). The sediment type was fine sand with silt. The interval 0 to 24 cm exhibited larger particle sizes, with more variation. From 24 cm to the base of the core, the particle size became finer with a size stable at ~3 Φ and mutations in some layers. The average total organic carbon (TOC) content was ~0.25%, and the average total nitrogen (TN) content was ~0.04%. The average TN increased from the surface to the base of the core. The TOC/TN ranged from 5.4 to 6.9, and the stable organic carbon isotope (δ^13^C-TOC) values ranged from −19.9 to −20.1‰. This showed mixed land–sea deposition. The age at the base of the core is 3395 yr before present (B.P.), indicating late Holocene strata.

Core ZC2 was recovered from the sand wave on the southern part of the shoal. The length of the core was 156 cm (153 cm under testing). The sediments were grey-black with black organic matter at the top and bottom. The sediment median particle size was 2.4–4.4 Φ (Fig. 7b), indicating silty sand and sandy silt. From 0–80 cm, the particle size gradually increased, and from 80 cm particle size decreased with the smallest particle (~4.4 Φ) at 100 cm; grain size then increased and the core showed changes in the rhythmic alternations. The average TOC was ~0.32% and average TN was ~0.05%. The TN showed variations; from the surface to the base of the core, it increased first, then decreased and then finally increased again. The TOC/TN was 5.2–7.5 and *δ*^13^C-TOC was −20.4 to −22.1‰, indicating a mixture of terrestrial and marine sediments. The age at the base of the core is 10806 yr B.P., indicating Holocene strata. Two cores revealed horizontal bedding and sand-mud interbed of structural characteristic^33^. Combined with age data and others research^34–36^, stratum frame of cores was normal sequence.

**Figure 8:**
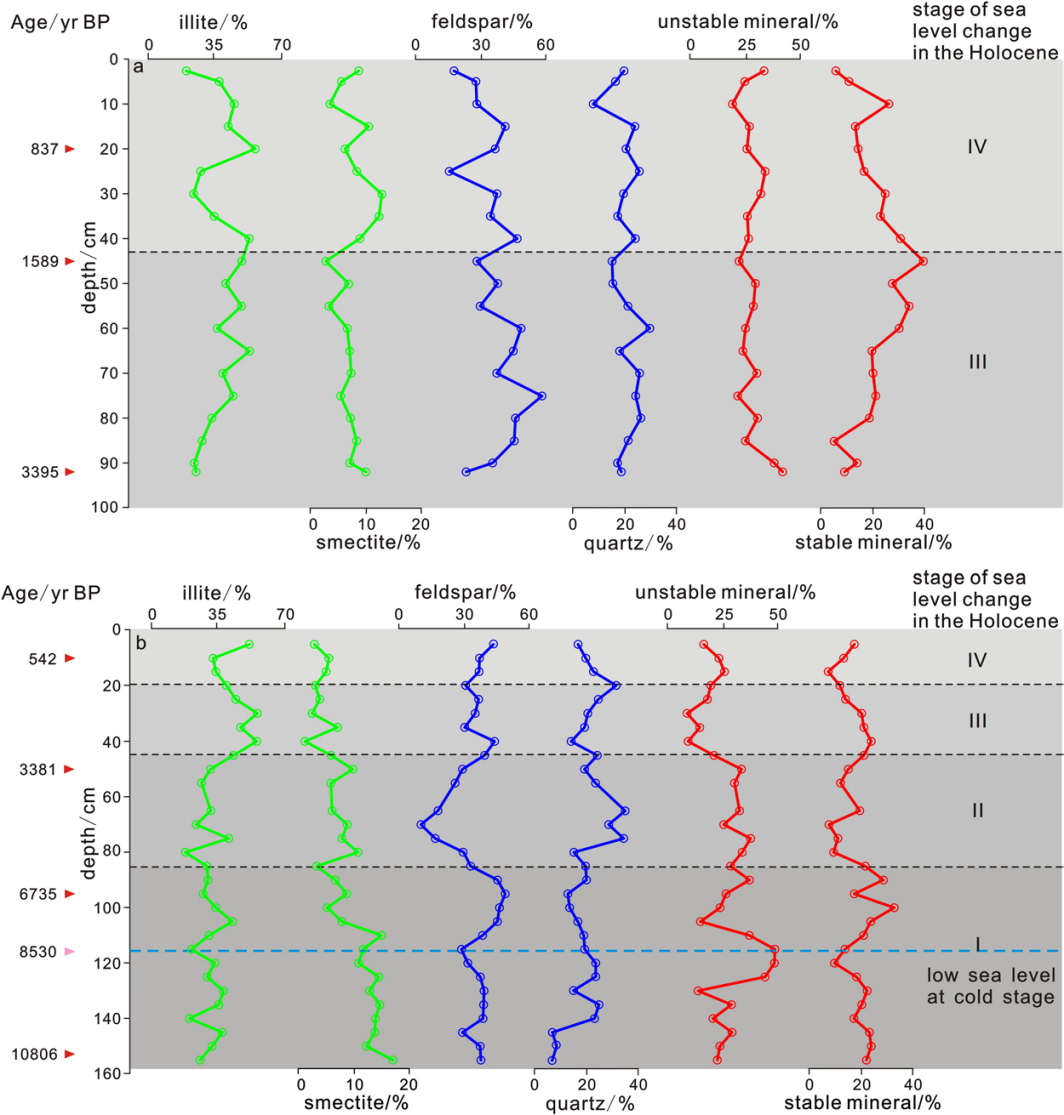
Main mineral profile of Core ZC1(**a**) and ZC2(**b**). The percentage content of typical mineral as illite, smectite, feldspar, quartz, unstable mineral and stable mineral are showed in this profile diagram.

Main mineral (illite, smectite, feldspar, quartz, unstable mineral and stable mineral) profiles were showed in Fig. 8. The content of main mineral in two profiles was similar with on Yangtze Shoal flat. The content of main mineral was displayed different variation trend in different stratum. These trends were also similar with TOC, TN and so on in Fig. 7.

## Discussion

The grain sizes, mineral components and organic matter content produced different mineral compositions and geochemical components. Sedimentary suspension and processes are a result of handling and flocculation and mainly controlled by the mechanical handling of the fluid^37–39^. Therefore, by tracing the physical and chemical components of the sediments, the sources can be traced and the formation and evolution of the sedimentary environment determined^40–42^.

The hydrodynamic conditions of the Yangtze Shoal are complex (Fig. 1). Because of the action of tidal currents (traveling at 1 to 2 kn) in the southeast, this energy is transferred southeast to northwest. The bottom flow of tidal current is the highest (velocity of 56 to 122 cm/s)^43^, with an ebb tide velocity of 55 to 70 cm/s. West of 124.5°E, the tidal direction is NW–SE to NNW–SSE, whereas east of 124.5°E the tidal current direction is NNW–SEE. The rotation of the tidal current over the Yangtze Shoal is very strong. In the area around 32°N and 124°E, the tide has a large ellipticity region and the tidal current ellipse is close to a circle. The tidal current velocities and ellipticity in the East China Sea shelf are both high, which provides favourable conditions for shoal development^44–46^. In addition to the effects of tidal currents from the western North Pacific, the study area is also affected by the Yellow Sea warm current, northern coastal stream, Taiwan warm current, Kuroshio Current and the Yangtze River diluted flow^7^. The Yellow Sea warm current and currents along northern Jiangsu carry with sediments, which are deposited in the northern and middle (eastern) part of the study area under the action of rotational flow. The sediments are enriched under the warm currents, Kuroshio Current and the Yangtze River outflow during different seasons in the study area^47^. The suspended sediment from the Yangtze River is mainly dispersed into the sea west of the Taiwan warm current and south of 32°N; the suspended sediment from the Yellow River is mainly input to the east of the Taiwan warm current, north of 32°N^48,49^. Therefore, source characteristics from northern Yangtze Shoal and the surface sediments of core ZC1 show a Yellow River signature, and estuary, southern Yangtze Shoal and surface sediments of core ZC2 demonstrate a Yangtze River source^50^.

**Figure 9:**
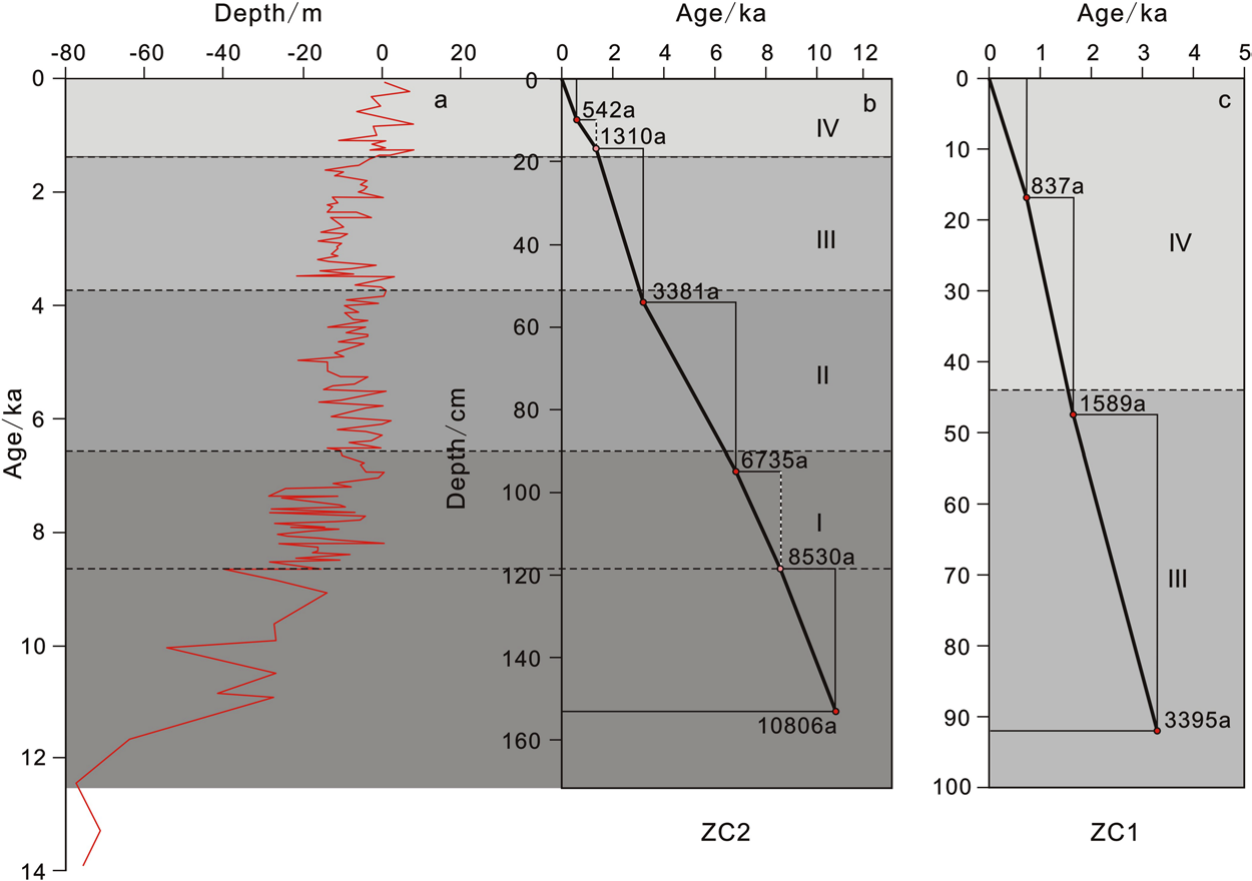
Sea level change and Core ZC1 (**c**), ZC2 (**b**) of sedimentary rate over the Holocene (compared with Grant, 2014, **a**). Since the Holocene, the Yangtze Shoal and surrounding area (East China Sea shelf) experienced two sea rises(II and IV stage) and two sea regressions(I and III stage) in sea level. The sedimentary rate of Core ZC1and ZC2 increased during the low sea level, and decreased during the high sea level.

Since the Holocene, the Yangtze Shoal and surrounding area (East China Sea shelf) experienced two rises in sea level and two regressions (Fig. 9)^51–54^, corresponding to global sea level changes, the Yangtze Shoal provenance and geological evolution exhibit a regional response to global sea level changes^55–58^.

Stage I persisted from 8300 to 6300 yr B.P. During this period, the strong winter monsoon in East Asia caused a certain degree of regression, corroborated from corresponding records from Greenland ice cores, and Dong Ge cave stalagmites^59–62^. Also during this period, the surface of the Yangtze Shoal was exposed, sedimentation increased with deposition of material supplied from the Yellow and Yangtze rivers. However, the Yangtze River had higher outflow than the Yellow River system that resulted in a greater contribution to the mixed sediments.

The second stage spanned 6300 to 3800 yr B.P. During this period of the Holocene, the monsoon climate was suitable, sea level rose, the eastern part of the Yangtze Shoal was inundated by seawater, and deposition decreased^60^. While the sediments remained a mixed source from the Yellow and Yangtze rivers, the material brought by the Yellow Sea warm current increased so that sedimentation was dominated by the Yellow River.

The third stage spanned 3800 to 1400 yr B.P. during which the overall intensity of the winter monsoon was lower with obvious fluctuations, indicating the instability and the drastic change of the monsoon climate^61^. This represented a synchronization of climate change in the Yangtze Shoal area and the wider East China Sea continental shelf. During this period, sea level was lower and part of the southern Yangtze Shoal was exposed. The sedimentation rate increased and the material exhibited a mixed source dominated by the Yangtze River.

After 1400 yr B.P. (the fourth stage), sea level rose and the entire Yangtze Shoal was inundated by seawater; the sedimentation rate subsequently decreased. As a result of the influence of a small ice age event, the Yangtze and Yellow rivers alternately dominated the sediment mixture, which caused the rhythmicity in the sedimentary column^63,64^.

**Figure 10:**
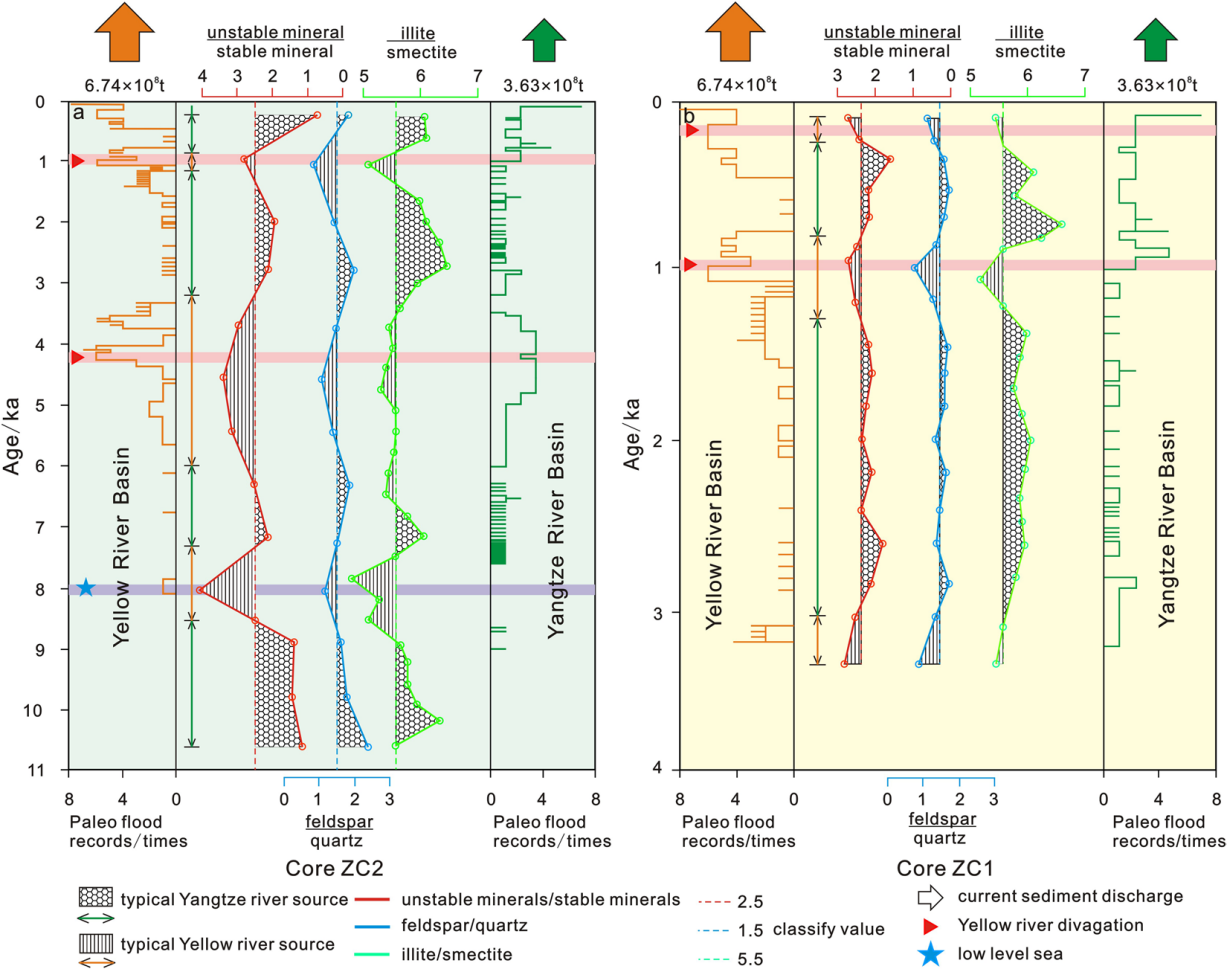
Compared with mineral contents of Core ZC1 (**b**) and Core ZC2 (**a**) and records of floods and sediment input of the Yangtze River and the Yellow River over the Holocene (Compared with Zhou and Yu, 2013). The ancient flood events were mainly concentrated in the Yangtze River basin over three periods: 7.5–6.5 ka, 5.0–3.5 ka and 1.0–0.5 ka. In the Yellow River basin, the flood events were mainly concentrated in 4.2–2.8 ka and 1.5–0.8 ka. This is consistent with the provenance of the detrital and clay minerals. Data of sediment discharge of Yangtze River and the Yellow River cites http://www.mwr.gov.cn/sj/. Paleo flood records refer to Zhou^68^.

The Yangtze and Yellow rivers experienced many river channel changes and flood events during the Holocene. The ancient flood events were mainly concentrated in the Yangtze River basin over three periods: 7.5–6.5 ka, 5.0–3.5 ka and 1.0–0.5 ka. In the Yellow River basin, the flood events were mainly concentrated in 4.2–2.8 ka and 1.5–0.8 ka (Table 1, Fig. 10)^65^. In addition, the Yellow River captured the Huai River into the sea at 4.2 ka, 1.0 ka and 0.2 ka, which provided a large amount of terrigenous material into the northern Jiangsu and Yangtze Shoals. The high frequency of ancient floods normally increases the sediment input into the sea. The amount of sediment from the Yangtze River entering the sea at 8.0–7.0 ka and 4.0–2.0 ka dropped sharply^66^. Coincident with this, flood events were frequent in the Yellow River and the amount of sediment entering the sea increased sharply. Therefore, the period of high sediment discharge from the Yangtze River occurred at 7.5–6.5 ka, 5.0–3.5 ka and 1.0–0.5 ka. For the Yellow River, high sediment discharge occurred at 8.0–7.0 ka, 4.2–2.8 ka and 1.5–0.8 ka. This is consistent with the provenance of the detrital and clay minerals^67–69^.

**Table 1:** Sedimentary types and input mechanism of the southern Yangtze Shoal in the Holocene.

Periods	Sea level/recent^48^	Ancient flood events	Yellow River divagation	Terrestrial material	Sedimentary types	Input mechanism
0–1.4 ka	high 0 m	Yangtze River frequent(1.4–0.8 ka) Yellow River frequent(1.0–0.5 ka)	0.2 ka and 1.0 ka	Yellow River source increase(0.2 ka,1.0 ka)	Mixed Yellow River(0.2 ka,1.0 ka)	Current
1.4–3.8 ka	low −10 m	Yellow River frequent(3.8–2.8 ka)	—	Yangtze River source increase	Yangtze River	Runoff and current
3.8–6.3 ka	high 0 m	Yangtze River frequent(5.0–3.8 ka) Yellow River frequent(4.2–3.8 ka)	4.2 ka	Yellow River source increase	Yellow River	Current
6.3–8.3 ka	low −20 m	Yangtze River frequent(7.5–6.5 ka) Yangtze River reduce(8.0 ka)	—	Yellow River source increase(8.0 ka)	Yangtze River Yellow River(8.0 ka)	Runoff and current
8.3–10.8 ka	low <−20 m	—	—	Yangtze River source increase	Yangtze River	Runoff

## Conclusions

The sediment types found in the area around the Yangtze Shoal showed zonal distribution. The area around the Yangtze Estuary is dominated by silty clay (6–8 Φ), with mainly fine sand (3–6 Φ) on the shoal. The grain size of the sediment in the grooves was small, and relatively large in the sand ridges or beach surface, consistent with the groove distribution trend. The core sediments changed in grain size from the top to the bottom of the cores with alternations of fine-coarse-thin-coarse layers. For surface sediments on the Yangtze Shoal, the illite content was high (average 62%), followed by smectite (17%), this is a typical characteristic of the Yangtze River terrigenous supply. Terrigenous material from the Yellow River was mainly distributed on the northern and western part of the shoal.

The sedimentary sequence of the beach and trenches on the southern Yangtze Shoal showed rhythmic thickness changes, indicating a change in multi-period hydrodynamic conditions as a result of the balance between transgression and regression and river recharge. The results of ^14^C dating showed that the base of Core ZC2 (trough) was ~10.8 ka (Holocene). The age of the base of Core ZC1 (ridge) was 3.36 ka, corresponding to the mid-late Holocene, indicating that the sedimentation rate was higher at ZC1. The slower sedimentation rate in the trough also indicates that the beach ridge in the study area is dominated by erosion–deposition processes (material re-transport), and the trench is dominated by slow and stable sedimentation. The clay and detrital mineral analysis showed different values and zoned results of smectite to illite and chlorite (S/(I + C)) that indicate sediments from different intervals mainly originated from the suspended material of the Yellow or Yangtze rivers. The mineral composition of amphibole, emerald, limonite and magnetite also indicates the same source; the clay minerals were in agreement with grain size changes. This comprehensive comparison of sediment grain size, clay and detrital minerals, and ^14^C dating in core samples indicated the source of sediment and changes in hydrodynamic conditions.

During the Holocene, the material of the southern Yangtze Shoal mainly originated from the Yangtze and Yellow rivers. Under the influence of sea level changes and the amount of riverine sediment supply, the deposition characteristics of the Yangtze Shoal consists of a mixed source from the Yangtze River, which is predominant; the contribution from the Yellow River increased during specific periods. During the periods 6.0–4.0 ka and 1.5–0.8 ka, sea level rose and the Yellow River was diverted southwards because of flood events. Material from the Yellow River was supplied to the Yangtze Shoal through coastal currents, resulting in increased contribution from the Yellow River on the shoal. During times of lower sea level, parts of the Yangtze Shoal were exposed and received recharge from the paleo-Yellow and Yangtze rivers. While the material input from the Yangtze River drastically reduced at 8.0 ka, the material from the Yellow River increased. During all other studied intervals, material from the Yangtze River dominated.

## Methods

A total 261 surface samples were used in this study with 111 samples collected during two cruises by the Science III (Open Cruise of National Science Foundation of China) on the southern part of the Yangtze Shoal in June 2013 (n = 79) and July 2015 (n = 32, Fig. 2). The remaining surface samples were collected by a fishing vessel (NO. 34002) in June 2013 (n = 15) and August 2014 (n = 12). Other surface samples were collected by “908” sedimentary survey^27^. Additionally, two sediment cores ZC1 and ZC2 were retrieved by another fishing vessel (NO. 79423) in August 2014. The cored sediments are dominated by continuous homogenous grey clay or silt with or without visible bioturbation. Samples were taken at 2 cm or 4 cm intervals from ZC1 and ZC2, respectively. Both surface and core sediments were used for multiple analyses in this study.

### Particle size

Grain size distribution of terrigenous particles was measured on bulk sediments with a Malvern Mastersizer 2000 Particle Size Analyzer at the Third Institute of Oceanography after removing carbonate and organic matter. Bulk sediments were treated successively with 10% H_2_O_2_ and 0.1 N HCl to remove organic material and carbonate^30^, respectively. A Shepard method was applied to calculate mean grain size (Mz), standard deviation (σi), skewness (SKi) and kurtosis (Kg), following the formulas utilized at the Third Institute of Oceanography.

### Detrital minerals

Bulk sediments were sieved and heavy minerals separated from the 63–125 μm fraction using bromoform (CHBr_3_) with a density of 2.89 g•cm^−3^. Both light and heavy minerals were analysed at Test central of minerals and rocks. Light minerals were mixed with epoxy (Epoxy-TEOA, 6:1), mounted and dried at 70 °C for 48 h, they were identified and counted using an optical microscope. For the heavy mineral analysis, a total of 300–500 particles were identified, and the sum and percentage of each species counted^70^.

### Clay minerals

Clay mineralogy was analysed by X-ray diffraction (XRD) using a PANalytical X’Pert PRO diffractometer at the State Key Laboratory of Marine Geology (Tongji University) on oriented mounts of carbonate-free clay-sized particles (<2 μm)^32,33^. All the samples were measured 3 times under air-dry conditions after ethylene glycol solvation for 24 h and heating at 490 °C for 2 h. Identification and interpretation of clay mineral species were made according to the (001) basal reflections on three XRD diagrams. The proportions of clay minerals in the assemblage were calculated semi-quantitatively using the MacDiff software (Petschick, 2000), based on peak areas of basal smectite reflections, including mixed layers (15–17 Å), illite (10 Å) and kaolinite/chlorite (7 Å), of the glycolated curve. The relative proportions of kaolinite and chlorite were calculated according to the ratio of the 3.57/3.54 Å peak areas. Replicate analysis of a few selected samples gave a precision of ±2% (2σ). The semi-quantitative evaluation based upon the XRD method had an accuracy of 5% for each clay mineral species.

### TOC and TN content and isotopic values

A Thermo NE1112 CN Elemental Analyzer connected to a Delta Plus AD Isotope Mass Spectrometer via Conflo III was used for sample analysis online. The elemental analysis furnace was set to 1020 °C, the reduction furnace to 650 °C and the column temperature to 40 °C^31^. Laboratory cylinders of CO_2_ and N_2_ were calibrated with the standards USGS-24, GBW4408 and IAEA-N1. Carbon and nitrogen isotopes are referenced to Pee Dee Belemnite (PDB) and atmospheric nitrogen, respectively. Laboratory determination accuracy was ±0.2‰. The measurements were conducted at the Third Institute of Oceanography (State Oceanic Administration).

### AMS ^14^C dating

Approximately 15 g of sample were processed for micropaleontological identification. Each sample was fully soaked, washed over standard 250 mesh (0.063 mm) sieve and dried. The retained material was then floated with carbon tetrachloride to concentrate any microfossils, isolated specimens were identified under a binocular microscope. In general, a minimum of 300 foraminifera were counted for quantitative analyses^22^ and selected foraminifera were sent to the United States Beta laboratory for ^14^C analyses. The age model of core was established using planktonic foraminifera (G. *ruber*) combined with eight AMS 14C data (Table 2). These AMS 14C dates were analyzed at the Beta Analytic Laboratory (USA) and were converted to calendar years by using the CALIB 7.0 program, with a 400-year reservoir correction and intercept of radiocarbon age with calibration curve. The result (S1) shows an age of ~10806a BP at the bottom (1.56 m) of the core.

**Table 2:** AMS 14 C data.

Core	Number of Beta	Depth (cm)	Conventional ^14^C Age (a B.P.)	Calibrated Age (yr B.P.)	material
ZC1	408770	20	810 ± 10	837	foraminifera(*G. ruber*)
	408771	45	1570 ± 30	1589	foraminifera(*G. ruber*)
	408772	92	3365 ± 30	3395	foraminifera(*G. ruber*)
ZC2	408773	10	530 ± 10	542	foraminifera(*G. ruber*)
	408774	50	3375 ± 10	3381	foraminifera(*G. ruber*)
	408775	95	6720 ± 30	6735	foraminifera(*G. ruber*)
	408776	120	8530 ± 50	8530	foraminifera(*G. ruber*)
	408857	153	10790 ± 50	10806	foraminifera(*G. ruber*)

## Electronic supplementary material

Supplementary Information
