# Supplementary material for: Temporal and spatial characteristics of sediment sources on the southern Yangtze Shoal over the Holocene
Source: Sci Rep. 2018 Oct 22;8:15577. doi: 10.1038/s41598-018-33757-5 (PMC6197290; doi:10.1038/s41598-018-33757-5)

Supplemental Information

Title: Temporal and spatial characteristics of sediment sources on the southern Yangtze Shoal over the Holocene

Author information

**Affiliations**

*Third Institute of Oceanography, State Ocean Administration, Daxue Road178, Xiamen, 361005, P. R. China*

*Chao Cao*

*Third Institute of Oceanography, State Ocean Administration, Daxue Road178, Xiamen, 361005, P. R. China*

*Feng Cai*

*Third Institute of Oceanography, State Ocean Administration, Daxue Road178, Xiamen, 361005, P. R. China*

*Yongling Zheng*

*Third Institute of Oceanography, State Ocean Administration, Daxue Road178, Xiamen, 361005, P. R. China*

*Chengqiang Wu*

*Third Institute of Oceanography, State Ocean Administration, Daxue Road178, Xiamen, 361005, P. R. China*

*Huiquan Lu*

*Third Institute of Oceanography, State Ocean Administration, Daxue Road178, Xiamen, 361005, P. R. China*

*Jingjing Bao*

*Third Institute of Oceanography, State Ocean Administration, Daxue Road178, Xiamen, 361005, P. R. China*

*Quan Sun*

**Contributions**

Chao Cao designed the study, wrote the main manuscript and prepared all figures. Feng Cai contributed to the improvement of the manuscript design and guaranteeing the quality of the manuscript. Yongling Zheng, Jingjing Bao processed the data. Chengqiang Wu, Huiquan Lu and Quan Sun collected the data. All authors reviewed the manuscript.

**Corresponding author**

Correspondence to Feng Cai

**Certificate of English Editing**

The English language in our manuscript was edited.


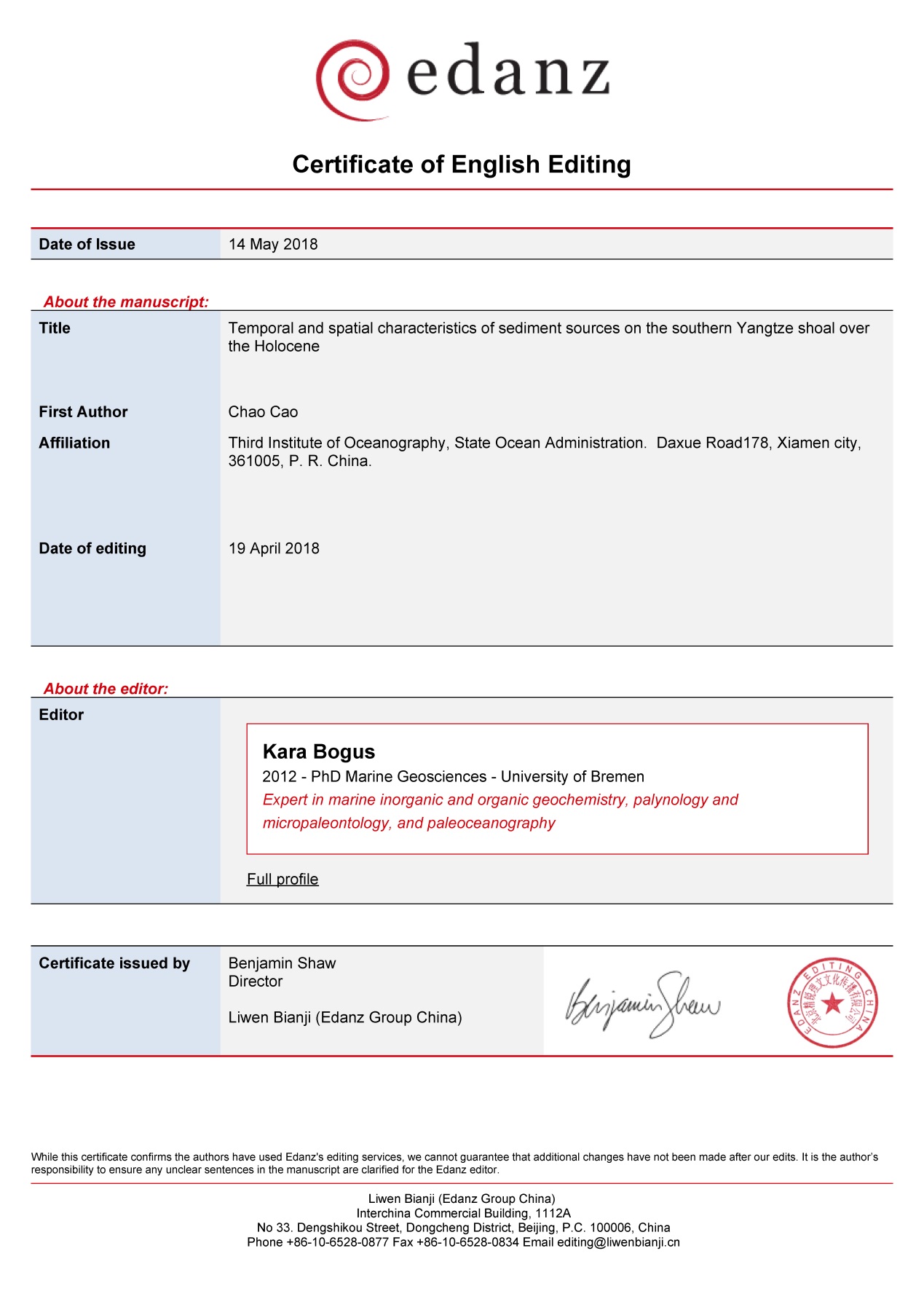

Supplement: Supplementary file 1 — Supplementary Information [file 41598_2018_33757_MOESM1_ESM.docx]
